# High-speed electro-optic modulator with group velocity matching on silicon substrate

**DOI:** 10.3389/fbioe.2025.1626017

**Published:** 2025-06-30

**Authors:** Yingbo Liu, Haiou Li, Yue Li, Haisheng Li, Yuxiang Hao, Liangpeng Qin, Jiayu Yang

**Affiliations:** ^1^ Guangxi Key Laboratory of Precision Navigation Technology and Application, Guilin University of Electronic Technology, Guilin, China; ^2^ The 34th Research Institute of China Electronics Technology Group Corporation, Guilin, China

**Keywords:** electro-optic modulators, TFLN, communication systems, group velocity, silicon substrate

## Abstract

Electro-optic modulators with low driving voltage and wide bandwidth are critical for advanced analog and digital communication systems. To achieve high-speed thin-film lithium niobate (TFLN) electro-optic modulators on silicon substrates, this work proposes a hybrid-loaded T type-U type traveling-wave electrode structure (TU-TWEs). The core of TU-TWEs in the introduction of an inductance compensation mechanism, which can effectively reduce the microwave refractive index and weaken the “slow light” effect, thereby matching the group velocity of light with the microwave velocity. We demonstrate a high-speed TFLN electro-optic modulator on a silicon substrate, with an electro-optic bandwidth greater than 110 GHz and a half-wave voltage of 1.35 V.

## 1 Introduction

The exponential growth of information technology has intensified the need for high-speed data transmission. Electro-optic modulators, being pivotal components for electrical-to-optical signal conversion in optical communications, and its performance enhancement is crucial for the realisation of high-speed and high-capacity optical communication systems (Winzer et al., 2018; Qi and Li, 2020) Nevertheless, traditional bulk lithium niobate modulators face limitations in size and integration level, failing to meet the dual demands of miniaturization and high performance in contemporary optical interconnection systems. TFLN renowned for its superior electro-optic effects, has emerged as a highly promising material for electro-optic modulator applications in recent years. The development of TFLN electro-optic modulators primarily focused on process exploration before 2018, where the maturity of etching and bonding techniques critically deter-mined device performance and stability (Rao et al., 2016; Hsu et al., 2017).

Marko Lončar’s team pioneered a CMOS-compatible TFLN electro-optic modulator in 2018 (Wang et al., 2018), achieving integrated devices with low half-wave voltage (Vπ) and high modulation efficiency through optimized etching and bonding processes. This mile-stone signified the evolution of TFLN modulators from process exploration to application development, laying the groundwork for future advancements. Nevertheless, conventional coplanar waveguide (CPW) electrode designs encounter critical limitations: high microwave losses, limited electro-optic bandwidth, and the mismatch be-tween microwave and optical group velocities is particularly prominent (Liu Y. et al., 2021). In response to this challenge, Mian Zhang et al. introduced a periodically loaded T-shaped micro-structured electrode design in 2021 (Kharel et al., 2021). mitigating microwave losses via in-creased electrode spacing while enhancing velocity matching through capacitive compensation strategies. Although this design achieved optimal microwave-optical velocity matching on quartz substrates, its primary constraint stems from substrate material limitations. The slow-light effect inherent to T-shaped electrodes substantially increases microwave refractive indices, rendering velocity matching unattainable on silicon substrates. Despite the advantages of low microwave loss with quartz substrates, challenges including prohibitive cost, mechanical fragility, and incompatibility with standard processes hinder their adoption for large-scale integration. Consequently, realizing effective microwave refractive index and optical group index matching on silicon substrates emerges as a pivotal challenge in developing low-cost, high-reliability TFLN modulators.

Present research into silicon-based group velocity matching concentrate on three principal approaches: 1. Silicon substrate etching: Reducing the equivalent microwave refractive index of silicon substrates through undercut etching (Chen et al., 2022) or backside etching (Wang M. et al., 2024). However, these methods rely on complex micro-nanofabrication processes with low process tolerance, inducing mechanical stress and waveguide distortions that degrade device yield.2. Optical delay tuning: Adjusting the effective refractive index of optical waves through waveguide design or phase compensation structures, such as the path delay design employed in cross-waveguide phase modulators (Du et al., 2024). While this approach avoids substrate etching, it requires precise control of optical path differences, features high design complexity, and tends to introduce addition-al insertion losses, making it unsuitable for mass production.3. Cascaded fast-slow wave electrodes (Wang S. Y. et al., 2024): This approach achieves equivalent group velocity matching through proportional adjustments, leveraging the alternating configuration of fast and slow wave traveling electrodes. This method has the potential for flexible design and process compatibility, but the issues of impedance matching and microwave reflection suppression need to be solved.

In summary, although implementing high-speed modulation on silicon substrates offers significant advantages, this process inevitably faces challenges including insufficient mechanical reliability, elevated optical losses, and etching difficulties. In response to these challenges, we propose a TFLN electro-optic modulator featuring a TU-TWEs. The design’s cornerstone is an inductive compensation mechanism that suppresses microwave refractive indices, alleviates “slow light” effects, and successfully enables optical group velocity-microwave velocity matching on silicon substrates, consequently enabling high electro-optic bandwidth. Through simulation verification, when the modulator length is 1 cm , the half-wave voltage of this structure is only 1.35 V ; at the same time, its electro-optic bandwidth is as high as 110 GHz , and the optical loss is as low as 0.08 dB/cm .

## 2 Principle of electro-optical modulators

The thin-film lithium niobate (TFLN) electro - optic modulator with a TU-TWEs proposed in this paper is composed of an optical part and an electrical part. The optical subsystem employs a Mach-Zehnder interferometer (MZI) configuration incorporating two multimode interference (MMI) splitters and parallel-aligned TFLN ridge waveguides. Input light is equally split into two coherent beams through the input MMI splitter, propagating through symmetrically arranged lithium niobate waveguides. TFLN’s pronounced electro-optic effect exhibits linear dependence of effective refractive index on external electric fields, enabling dynamic phase modulation of optical waves. Specifically, equal-magnitude counter-directional electric fields generated across vertical electrode gaps create +Δφ and −Δφ phase differences in respective waveguides. The phase-modulated optical beams recombine through output MMI interference, realizing intensity-modulated output through constructive/destructive interference (Tang et al., 2025). The change in refractive index of the optical material is expressed by Equation 1:

$$\Delta n=\frac{1}{2}\frac{n_{e}^{3}r_{33}}{n_{\mathrm{eff}}}\frac{V}{gap}\Gamma_{\mathrm{om}}$$ (1)

Where V is the modulation voltage, gap is the electrode spacing, $n_{e}$ is the extraordinary refractive index of lithium niobate, $n_{eff}$ is the effective refractive index, and $\Gamma_{om}$ represents the normalized overlap factor between electric and optical fields. The mathematical expression of $\Gamma_{om}$ is given by Equation 2 (Li et al. 2023):

$$\Gamma_{\mathrm{om}}=\frac{\iint_{S_{\mathrm{LN}}}\frac{E_{Z}\left(x,y\right)}{V}\cdot {\left|e_{Z}\left(x,y\right)\right|}^{2}dxdz}{\iint_{S}{\left|e_{Z}\left(x,y\right)\right|}^{2}dxdz}$$ (2)

$E_{z}\left(x,y\right)$ represents the electric field along the z-axis of lithium niobate from electrodes, $e_{z}\left(x,y\right)$ denotes the TE-mode optical field, $S_{\mathrm{LN}}$ is the cross-sectional area of the lithium niobate region, and S represents the entire modulator cross-section. The optical phase change is formulated through Equation 1 as Equation 3:

$$\Delta \phi =\frac{n_{e}^{4}r_{33}\pi V}{\lambda n_{eff}}\Gamma_{om}$$ (3)

The half-wave voltage ( $V_{\pi }$ ) serves as a key parameter, defined as the voltage to induce a π-phase shift in the optical wave. Reduced $V_{\pi }$ values correspond to enhanced modulator sensitivity. Typically proportional to device length, the modulation efficiency is quantified by the $V_{\pi L}$ product. For push-pull modulators, this product is expressed as Equation 4 (Pan et al., 2022; Hou et al., 2024; Liu et al., 2024):

$$V_{\pi }L=\frac{1}{2}\frac{\lambda n_{\mathrm{eff}}}{n_{e}^{4}r_{33}\Gamma_{\text{om}}}$$ (4)

Where λ is the optical wavelength, $n_{0}$ is the intrinsic refractive index of lithium niobate, $r_{33}$ represents the electro-optic coefficient of LN, and L is the length of the LN layer.

The three key factors limiting the bandwidth of the modulator are: (1) impedance matching; (2) microwave loss, primarily absorbed by the traveling waveguide and the silicon substrate; and (3) matching the group velocity of light with the microwave signal velocity.

1. Characteristic impedance matching refers to the state where the source impedance, transmission line characteristic impedance, and load impedance achieve equality. Impedance mismatch causes signal reflections at discontinuities along transmission lines, sending partial signals back to the source. Such reflections lead to signal attenuation and introduce interference, degrading overall signal quality.
2. Conductor loss is one of the primary losses in microwave transmission, mainly caused by the finite conductivity of metals. When current flows through metals, resistive losses occur due to inherent resistance. The height of metallic electrodes influences current flow paths and distribution patterns. High-frequency operation accentuates skin effect phenomena, confining current within the conductor’s surface layer. When electrode thickness falls below the skin depth threshold, current confinement to di-minished cross-sections increased resistive losses. Electrode height also affects electric field distribution within transmission lines. Improper electrode height may cause electric field leakage or radiation into unintended regions, generating additional radiative losses.
3. In traveling-wave electro-optic modulators, light propagates through lithium niobate waveguides while microwave signals travel along microwave guides. Mismatched group and phase velocities prevent uniform voltage modulation along the waveguide. At high modulation frequencies, this velocity mis-match introduces phase errors that degrade modulation performance.

Capacitively loaded traveling-wave electrodes (CL-TWEs) have been demonstrated to enhance electro-optic modulation bandwidth in TFLN electro-optic modulators (Nelan et al., 2022). The bandwidth improvement mechanism of CL-TWEs originates from two synergistic effects: 1) The T-type electrode enlarges inter-electrode spacing, mitigating current crowding effects and lowering microwave propagation losses. This design strategy is crucial for reducing the energy dissipation during the transmission process and helps to improve the overall performance and bandwidth of the modulator. 2) When implemented on quartz substrates, CL-TWEs simultaneously satisfy microwave-optical velocity synchronization and 50 Ω impedance matching. This dual matching condition minimizes signal reflections and phase distortions, significantly extending the 3- dB bandwidth ceiling.

## 3 Design and simulation

**FIGURE 1:**
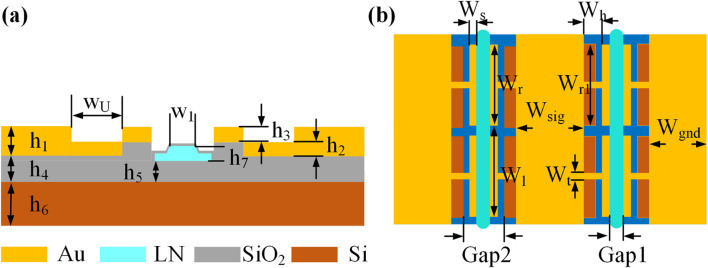
Schematic diagrams of the periodically hybrid-loaded T type-U type traveling-wave electrode structure TFLN electro-optic modulator: **(a)** Cross-section of the modulator, $h_{1}=1.8\mu m$ , $h_{2}=0.9\mu m$ , $h_{3}=0.9\mu m$ , $h_{4}=5.5\mu m$ , $h_{5}=4.7\mu m$ , $h_{6}=500\mu m$ , $h_{7}=0.4\mu m$ , $w_{1}=1.2\mu m$ , $w_{U}=4\mu m.$ **(b)** Top view of the modulator, $W_{S}=2\;\mu m$ , $W_{h}=6\;\mu m$ , $W_{r}=45\;\mu m$ , $W_{l}=50\;\mu m$ , $W_{t}=2\;\mu m$ , $W_{sig}=25\;\mu m$ , $W_{gnd}=200\;\mu m$ , ${Gap}_{1}=3\;\mu m$ , ${Gap}_{2}=11\;\mu m$ , $W_{r1}=45\;\mu m$ .

Figure 1a illustrates the cross-sectional structure of the modulator, comprising a 500 μm silicon substrate with 4.7 μm thermally grown silicon dioxide layer, a 400 nm thick layer of TFLN is bonded onto the silicon dioxide. The silicon dioxide layer on TFLN primarily functions as a protective cladding, serving the dual role of mitigating optical propagation loss and enhancing modulation efficiency. The TFLN is etched into ridge waveguide structures that provide stronger optical mode confinement and lower effective refractive indices. An optimized ridge waveguide width of 1.2 μm is implemented to balance optical confinement and propagation loss. Complete etching of the TFLN ridge waveguide is performed, retaining only the optical waveguide transmission region. These processes significantly enhance the electro-optic modulation efficiency. A metallic electrode layer is deposited above the TFLN, with the traveling-wave electrode structure depicted in Figure 1b. The metal electrode consists of three parts: the main electrode, the U type load structure, and the T type load structure. We subsequently derive the transmission line equivalent model of the TU-TWEs to analyze its impact on enhancing electro-optic modulation bandwidth performance. This design strategy achieves additional bandwidth expansion by simultaneously optimizing microwave propagation characteristics and impedance matching.

### 3.1 Analysis of transmission line equivalent circuit model

**FIGURE 2:**
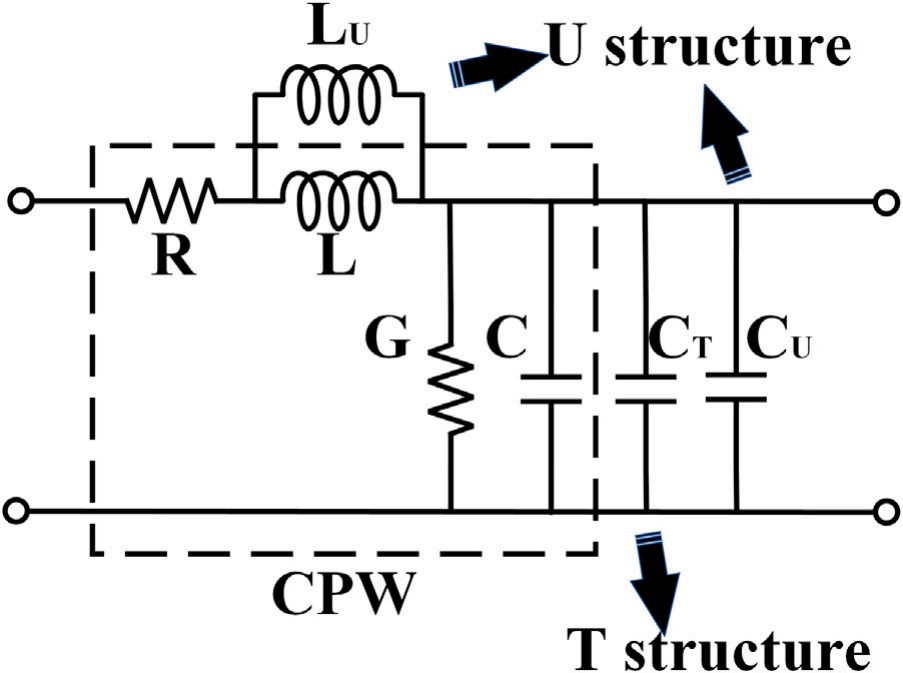
Equivalent circuit of hybrid-loaded T type-U type traveling-wave electrode structure (TU-TWEs).

The transmission line equivalent model of hybrid-loaded T type-U type travel-ing-wave electrode structure (TU-TWEs) is shown in Figure 2.

Based on microwave theory, the characteristic impedance and propagation constant formulations derived from the equivalent transmission line model are given by Equations 5, 6 (Fang et al., 2023; Cheng et al., 2024):

$$Z_{0}=\sqrt{\frac{R_{eff}+j\omega L_{eff}}{G_{eff}+j\omega C_{eff}}}$$ (5)

$$\gamma =\sqrt{\left(R_{eff}+j\omega L_{eff}\right)\left(G_{eff}+j\omega C_{eff}\right)}$$ (6)

Where $R_{eff}$ is the equivalent resistance, $G_{eff}$ the equivalent conductance, $C_{eff}$ the equivalent capacitance, and $L_{eff}$ the equivalent inductance of the transmission line. When analyzing frequency responses beyond 10 GHz , the lossless transmission approximation ( ωC/G≫1 ; ωL/R≫1 ) becomes valid. The expressions then reduce to Equations 7, 8:

$$Z_{0}=\sqrt{\frac{L_{eff}}{C_{eff}}}$$ (7)

$$\gamma =-\omega \sqrt{L_{eff}C_{eff}}$$ (8)

The microwave refractive index is formulated through the propagation constant Equation 9:

$$N_{p}=\frac{c*IM\left(\gamma \right)}{\omega }$$ (9)

The equivalent inductance and capacitance extracted from Figure 2 model are expressed as Equations 10, 11:

$$L_{eff}=\frac{L_{U}+L}{LL_{U}}$$ (10)

$$C_{eff}=C+C_{T}+C_{U}$$ (11)

The equivalent inductance equals the parallel combination of parasitic inductances from main and U-type electrodes, while the equivalent capacitance results from parallel parasitic capacitances of main, T-type, and U-type electrodes.

The U-type structure contributes only parasitic inductance without introducing parasitic capacitance. The velocity synchronization mechanism is achieved through: Compared to conventional CL-TWEs, the integrated U-type structure introduces parallel inductive loading that suppresses equivalent inductance, counterbalancing the microwave refractive index elevation induced by T-type capacitive loading. Although this design increases characteristic impedance as Equation 5, the impedance variation can be mitigated through optimized thickening of the underlying SiO_2_ layer (Li et al., 2024).

### 3.2 High frequency analysis of modulator

**FIGURE 3:**
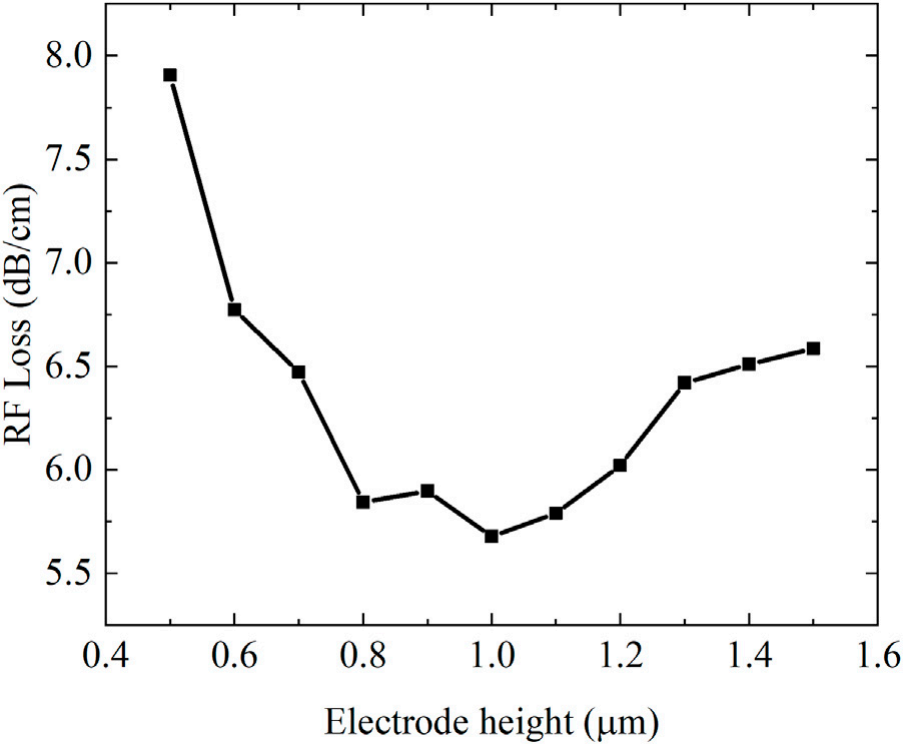
The trend of RFs Loss with the electrode height.

According to the above analysis, microwave loss is one of the critical factors limiting electro-optic bandwidth. The influence of modulator electrode height on device performance is analyzed via finite element method (FEM) simulations, calculating microwave losses at 75 GHz . Results shown in Figure 3 demonstrate that when electrode height is too small, the skin effect becomes particularly pronounced, confining current propagation predominantly to the conductor’s surface layer. Such concentrated distribution of current will increase the resistance and further significantly increase the loss during the microwave transmission process. When the electrode height is too large, it may have an adverse impact on the distribution of the electric field in the transmission line. Specifically, oversized electrodes may distort normal field distribution patterns, causing partial field energy leakage or radiative emissions. This unintended energy dissipation is termed radiation loss. Minimum microwave loss of 5.6 dB/cm at 75 GHz is achieved with 1 μm electrode height. Consequently, $h_{2}=h_{3}=1\;\mu m$ is selected as the optimal configuration for microwave loss minimization.

**FIGURE 4:**
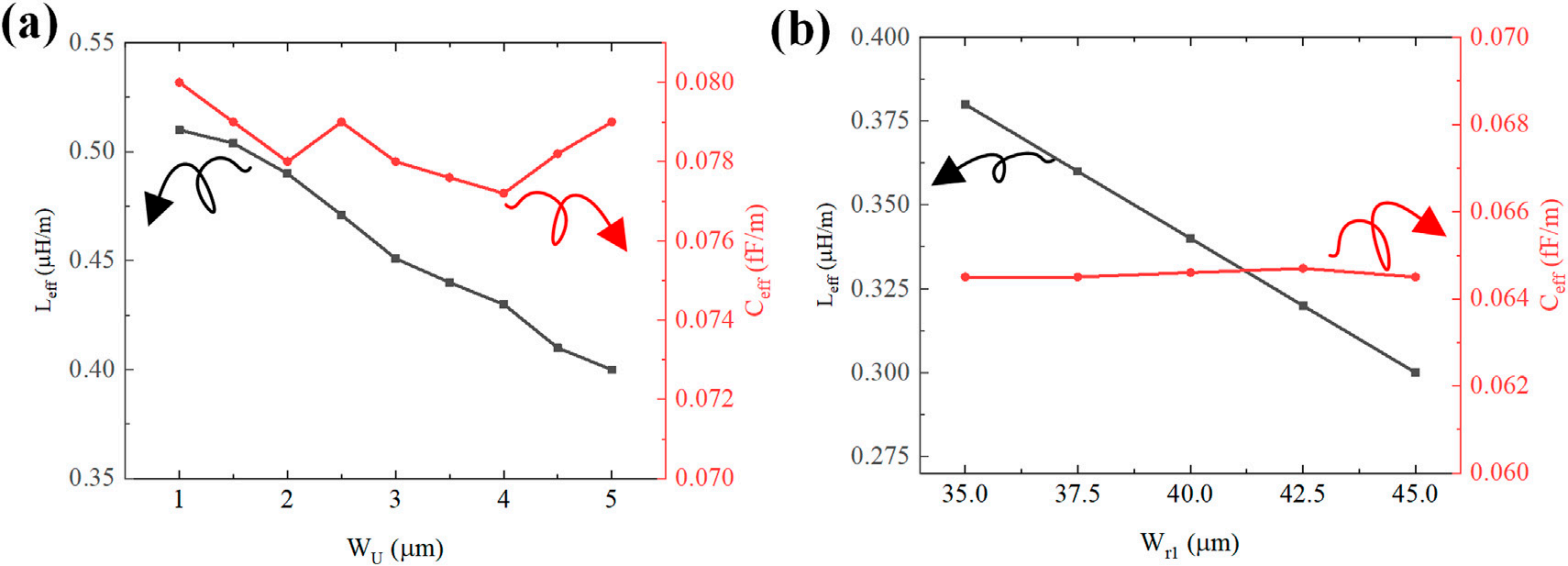
Dependence of equivalent circuit parameters with U-type structure: **(a)** Equivalent inductance and capacitance with $W_{u}$ ; **(b)** Equivalent inductance and capacitance with $W_{r1}$ .

Through systematic analysis of U-type structure’s influence demonstrates that parametric tuning of $W_{r1}$ and $W_{u}$ enables optimized inductance-capacitance distribution in the U-type configuration. As shown in Figure 4 simulations at 75 GHz , $W_{u}$ parameter adjustments exhibit limited capacitance variation but substantial inductance modulation. Specifically, increasing $W_{u}$ causes equivalent capacitance to fluctuate slightly around 0.078 fF/m , while equivalent inductance decreases from 0.5 nH/m to 0.4 nH/m ( 20% reduction). Similarly, increasing $W_{r1}$ maintains equivalent capacitance near 0.065 fF/m with minimal variation, while reducing equivalent inductance from 0.375 nH/m to 0.3 nH/m ( 25% reduction). Extensive simulations confirm the U-type structure’s effective inductance tuning capability with negligible capacitance influence. These findings establish the U-type component as an effective inductive compensation mechanism for microwave-optical velocity matching in modulator electrode design.

**FIGURE 5:**
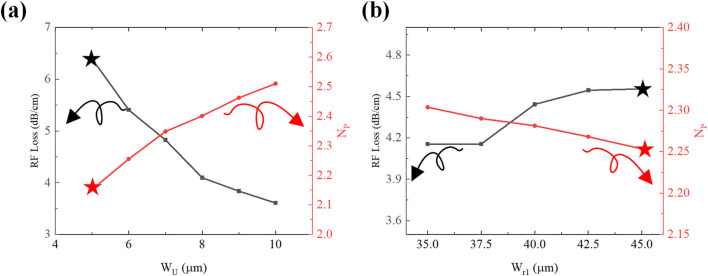
RF performance trends with U-type structure; **(a)** RF Loss and microwave refractive index trends with $W_{u}$ . **(b)** RF Loss and microwave refractive index trend with $W_{r1}$ .

We conducted an in-depth analysis of the U-type structure’s effects on microwave refractive indices and radiofrequency attenuation characteristics. Simulation results in Figure 5 show that increasing $W_{u}$ from 4 μm to 10 μm reduces RF loss at 75 GHz from 6.5 dB/cm to 3.5 dB/cm , while increasing microwave refractive index from 2.17 to 2.52 . At $W_{u}=4\;\mu m$ , optimal refractive index matching with optical group velocity ( 2.25 ) is achieved while maintaining low microwave losses. Consequently, $W_{u}=4\;\mu m$ is identified as the design optimum through comprehensive parametric analysis. Similarly, increasing $W_{r1}$ from 35 μm to 45 μm slightly elevates RF loss from 3.9 dB/cm to 4.5 dB/cm while marginally decreasing microwave refractive index from 2.32 to 2.25 . $W_{r1}=45\;\mu m$ is chosen to achieve optimal refractive index matching with optical group velocity.

While the T-type structure exhibits negligible impact on equivalent inductance, it substantially modulates equivalent capacitance values (Liu X. C. et al., 2021). The following analysis focuses on the effects of parameters $W_{S}$ , $W_{h}$ , $W_{T}$ , and $W_{R}$ on modulator speed and impedance characteristics.

**FIGURE 6:**
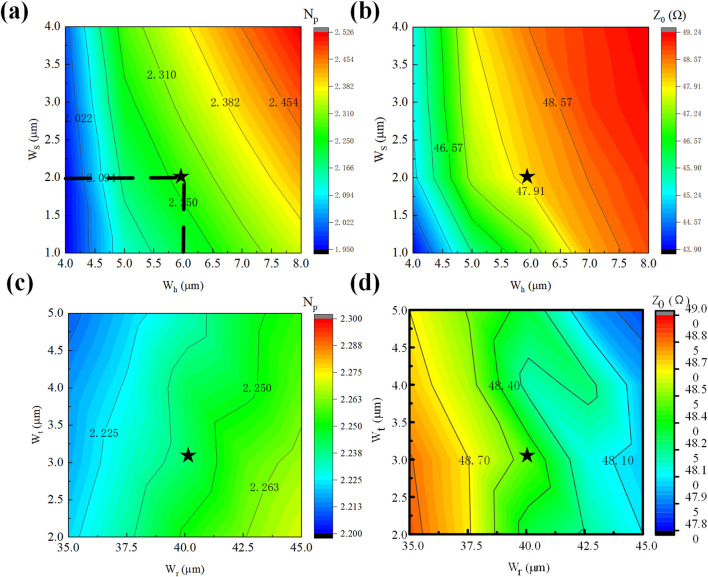
Trend of RF performance with T-type structure parameters $W_{S}$ and $W_{h}$ . **(a)** Contour thermogram of microwave refractive index with $W_{S}$ and $W_{h}$ . **(b)** Contour thermograms of characteristic impedance as a function of $W_{S}$ and $W_{h}$ .Trend of RF performance with T-type structure parameters $W_{r}$ and $W_{t}$ , **(c)** Contour thermogram of microwave refractive index with $W_{r}$ and $W_{t}$ . **(d)** Contour thermogram of characteristic impedance variation with $W_{r}$ and $W_{t}$ .

We conducted systematic parametric analysis of $W_{S}$ and $W_{h}$ dimensions to evaluate their effects on modulator characteristics. Figures 6a,b presents contour mapping results obtained through parametric optimization scanning. Both microwave refractive index and characteristic impedance increase with $W_{S}$ and $W_{h}$ dimensions. The configuration $W_{S}=6\;\mu m$ / $W_{h}=2\;\mu m$ achieves optimal microwave refractive index ( 2.27 ) and characteristic impedance of 48 Ω . The geometric parameters $W_{S}$ and $W_{h}$ critically alter the parasitic parameters in the transmission-line equivalent model, consequently modifying microwave propagation properties.

Experimental investigations reveal limited parametric influence on overall device characteristics. Figures 6c,d displays contour mapping results obtained through comprehensive parametric optimization. Increasing $W_{r}$ elevates microwave refractive index while reducing characteristic impedance, whereas $W_{t}$ increments decrease both mi-crowave refractive index and impedance. The configuration $W_{r}=40\;\mu m$ / $W_{t}=3\;\mu m$ is identified as optimal, delivering microwave refractive index 2.24 and characteristic impedance of 48.5 Ω .

### 3.3 Influence on modulation efficiency

Key determinants of electro-optic conversion efficiency include waveguide effective refractive index and electro-optic field overlap factor. Effective refractive index is governed by geometric parameters of the ridge waveguide, specifically ridge width and, etch depth. For defined waveguide architectures and operating wavelengths, the $V_{\pi L}$ product exhibits inverse proportionality to the overlap factor ( $\Gamma_{om}$ ), where enhanced field overlap reduces $V_{\pi L}$ and improved modulation efficiency. The electro-optic overlap factor is primarily determined by parameters such as electrode spacing and electrode position. Research indicates that enlarging the electrode spacing decreases the half-wave voltage-length product ( VπL ), thereby directly improving electro-optic efficiency. Nevertheless, decreasing the electrode spacing induces a substantial rise in optical absorption loss, requiring careful balancing of modulation efficiency and optical loss in the design process.

**FIGURE 7:**
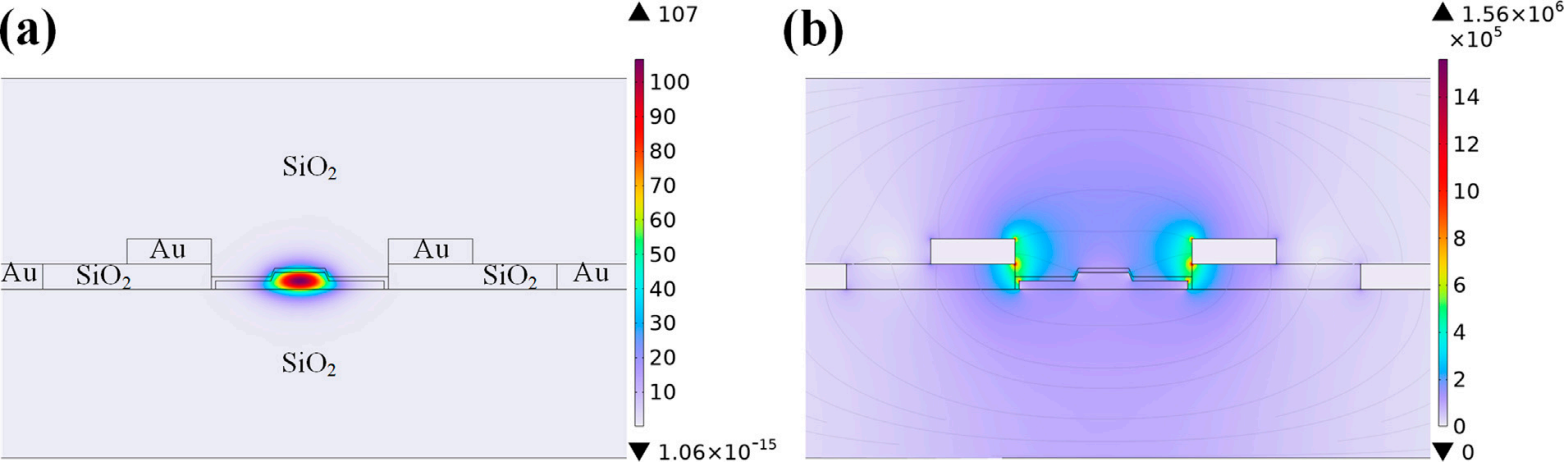
**(a)** TE mode optical field of the modulator. **(b)** Electrostatic field of the electrode.

Guided by the theoretical framework of the half-wave voltage-length product Equation 5, numerical simulations of the modulator’s optical mode field and electro-static field were conducted using finite element analysis software. The optical mode electric field distribution is depicted in Figure 7a, and the electrostatic field distribution is presented in Figure 7b.

**FIGURE 8:**
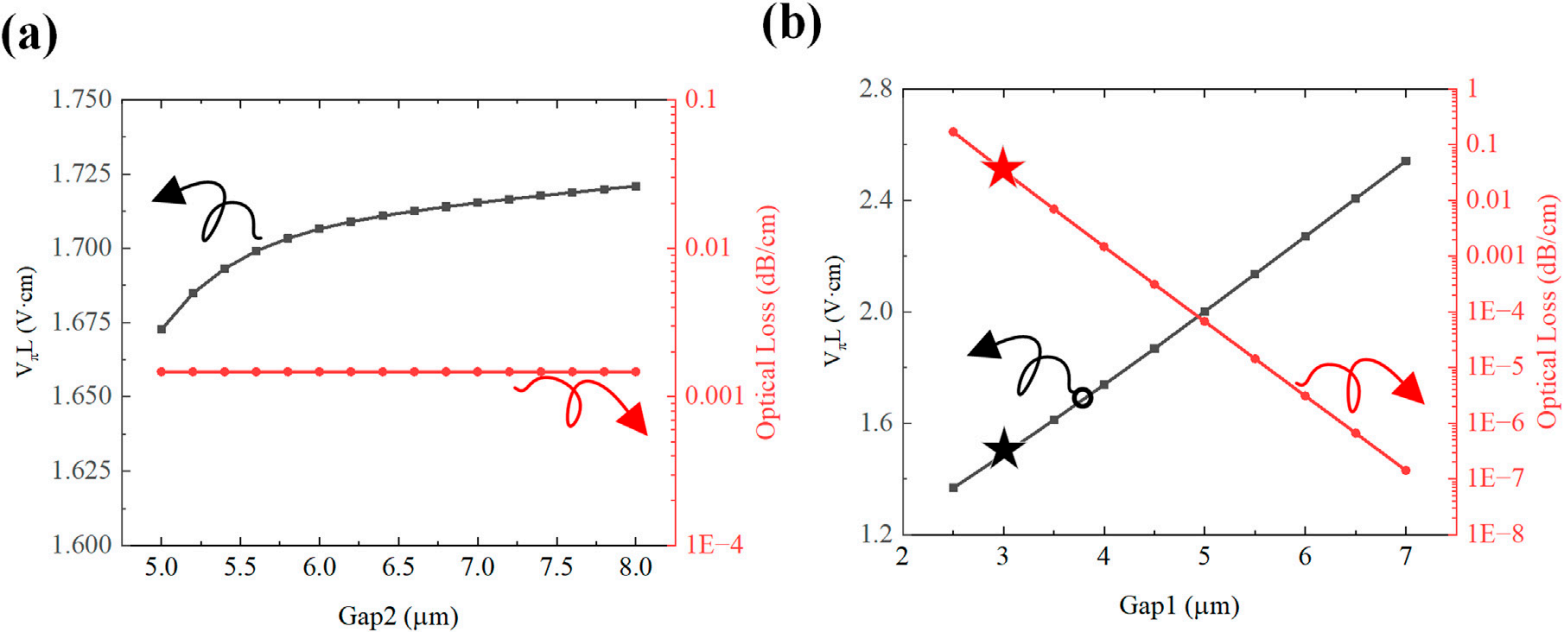
U-type structure and T-type structure electrode spacing on modulation efficiency **(a)** Trend of modulation efficiency and optical loss with U-type structure spacing Gap2 . **(b)** Trend of modulation efficiency and optical loss with T-type structure spacing Gap1 .

In the T-type structure, Gap1 exhibits a pronounced impact on modulation efficiency—reducing Gap1 lowers VπL and enhances modulation efficiency at the expense of elevated optical loss. In contrast, the U-type structure demonstrates limited sensitivity of modulation efficiency to variations in Gap2 . Simulation results in Figure 8a reveal that increasing Gap2 from 5 μm to 8 μm causes VπL to shift marginally from 1.66 V·cm to 1.72 V·cm , with negligible changes in optical loss. Gap2 is constrained by the geometric parameters $W_{h}$ and $W_{u}$ , while Gap1 optimization necessitates a compromise between optical loss and modulation efficiency. As demonstrated in Figure 8b, sweeping Gap1 from 2.5 μm to 7 μm leads to a monotonic increase in the half-wave voltage-length product from 1.3 V·cm to 2.5 V·cm . The optimal Gap1 value of 3 μm was chosen, achieving a balanced performance with a half-wave voltage of 1.35 V·cm and an optical loss of 0.08 dB/cm .

### 3.4 Electro-optic modulation bandwidth simulation

According to microwave transmission theory, the optical-electric transmission function can be expressed as Equation 12 (Jing et al., 2020):

$$\mathrm{HF}\left(\omega \right)=20\log_{10}\left\{\frac{2\sqrt{Z_{0}Z_{in}}}{Z_{0}+Z_{in}}e^{-\frac{\alpha L}{2}}\sqrt{\frac{\sin h^{2}\left(\frac{\alpha L}{2}\right)+sin^{2}\left(\frac{bL}{2}\right)}{{\left(\frac{\alpha L}{2}\right)}^{2}+{\left(\frac{bL}{2}\right)}^{2}}}\right\}$$ (12)

The propagation loss α the deviation between optical and microwave speeds expressed as b , the total length of the modulator L , the input impedance typically set to 50 Ω , the characteristic impedance of the transmission line $Z_{0}$ , and the speed matching term $b=\frac{2\pi f}{c}\left(n_{m}-n_{g}\right)$ are key parameters in determining the modulator’s performance. Equation 1 provides a unified formulation to evaluate the impact of impedance matching, speed matching, and loss on optical efficiency.

**FIGURE 9:**
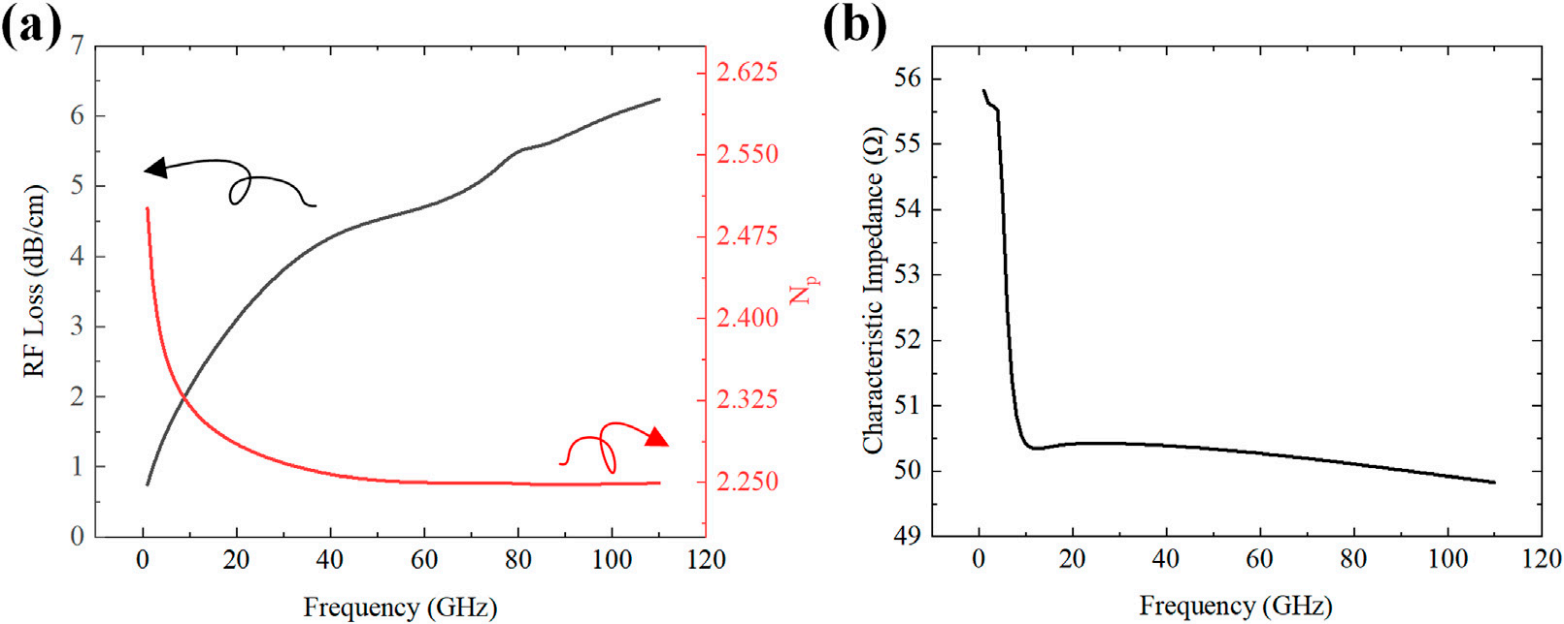
RF performance of the modulator **(a)** Frequency response of RF Loss and microwave refractive index. **(b)** Frequency response of characteristic impedance.

We performed a comprehensive finite-element analysis of the modulator’s performance. We specifically calculated its characteristic impedance, microwave loss, and microwave speed as key parameters. In this study, the modulator length was set to 1 cm , corresponding to a half-wave voltage of 1.35 V . We performed a comprehensive finite-element analysis of the modulator’s performance. The simulation frequency range is from 1 to 110 GHz , and the corresponding microwave loss and microwave refractive index are shown in Figure 9a. Within the entire frequency range, the microwave loss remains below 6.2 dB/cm , while the microwave refractive index gradually approaches 2.25 at higher frequencies. Additionally, we calculated the S parameters to obtain the ABCD matrix and derived the device’s characteristic impedance from this matrix. The results are shown in Figure 9b. As shown in the figure, the device’s characteristic impedance primarily fluctuates around 50 Ω .

**FIGURE 10:**
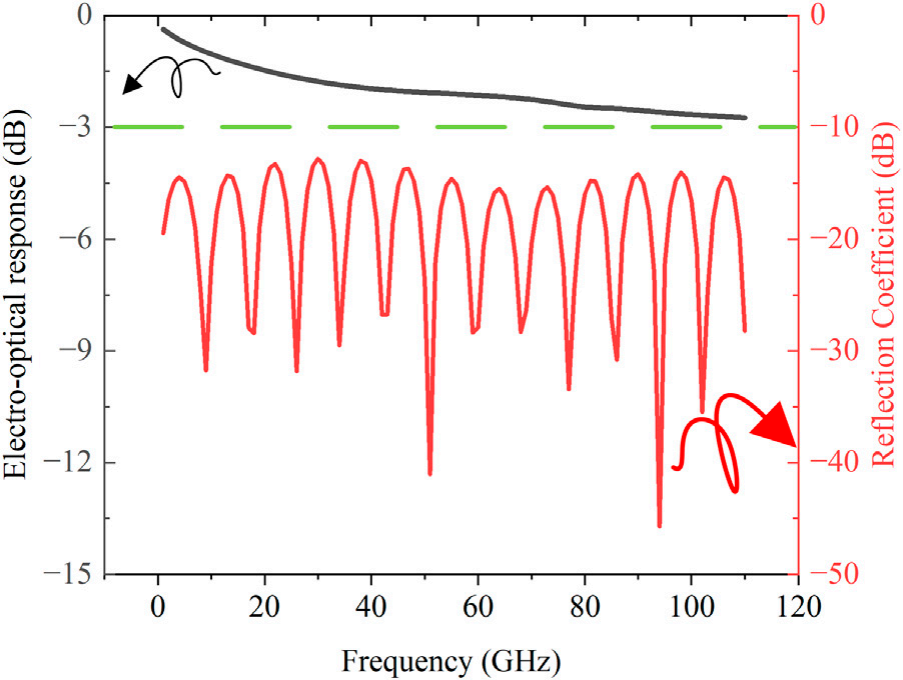
Electro-optic response and reflection coefficient of a 1cm -long modulator.

Finally, by substituting the calculated frequency-dependent microwave loss, refractive index, and characteristic impedance of the device into Equation 1, the electro-optic response shown in Figure 10 was obtained. Simulation results demonstrate a −2.8 dB rolloff in the electro-optic response at 110 GHz , with the reflection coefficient maintained below −10 dB across the operational bandwidth.

Through simulations and analysis, we demonstrated a thin-film lithium niobate electro-optic modulator chip with a half-wave voltage of 1.35 V and an electro-optic response bandwidth exceeding 110 GHz , demonstrating its exceptional electro-optic bandwidth performance. We have summarized the modulator’s performance in Table 1 and conducted comprehensive comparisons with state-of-the-art technologies. This table demonstrates that our work achieves high electro-optic bandwidth on silicon substrate through simulation verification, and successfully realizes a modulator structure design with good matching between optical group velocity and microwave velocity.

**TABLE 1:** Performance Comparison of LNOI MZI Modulator.

	$V_{\pi }$ (V)	Length (mm)	Optical loss (dB/cm)	S_11_ (dB)	EO bandwidth (GHz)
[6]	1.4	20	0.2	-	45^b^
[7]	1.75	5	0.7	-	>40^b^
[8]	2.3	10	-	<-15	>100^b^
[9]	2.2	10	0.2	<-25	>67^b^
[10]	2.18	10	-	<-20	>67^b^
[11]	1.85	4	0.39	<-10	>67^b^
Our work	1.35	10	0.08	<-10	>110^a^

^a^ : Simulation Results.

^b^ : test result.

## 4 Conclusion

In this work, we present a high-speed TFLN electro-optic modulator on a silicon substrate that achieves a match between microwave velocity and optical group velocity. Specifically, we design and propose a TU-TWEs. The introduction of the U-type electrode in this structure effectively reduces the equivalent inductance of the transmission line without significantly affecting its equivalent capacitance. This unique inductance compensation mechanism helps to mitigate the “slow light” effect, enabling a good match between the optical group velocity and microwave velocity on a silicon substrate and significantly enhancing the electro-optic bandwidth. Simulation results show that when the modulator length is 1 cm , the half-wave voltage of this structure is only 1.35 V ; meanwhile, its electro-optic bandwidth is as high as 110 GHz , and the optical loss is as low as 0.08 dB/cm . Additionally, the traveling-wave electrode structure we propose has high scalability and can be hybrid-integrated with high dielectric constant materials (such as barium titanate, etc.) or heterogeneously integrated with silicon photonic chips in the future to achieve even more outstanding modulation performance.

## Data Availability

The original contributions presented in the study are included in the article/supplementary material, further inquiries can be directed to the corresponding author.
